# Breast Cancer in Adolescents and Young Adults Less Than 40 Years of Age in Nigeria: A Retrospective Analysis

**DOI:** 10.1155/2022/9943247

**Published:** 2022-07-29

**Authors:** Atara Ntekim, Mojisola Oluwasanu, Oluwaponmile Odukoya

**Affiliations:** ^1^Department of Radiation Oncology, College of Medicine, University of Ibadan, Nigeria; ^2^Department of Health Promotion, College of Medicine, University of Ibadan, Nigeria

## Abstract

**Background:**

Breast cancer among adolescents and young adult (AYA) females aged 15-39 years is associated with different patterns of aggressiveness, as well as psychosocial and economic issues. At present, the burden of breast cancer among this age group is unknown in Nigeria. There is a need to determine the proportion of AYA with breast cancer in Nigeria. This will inform the development of breast cancer care programs appropriate for this age group.

**Objective:**

The objective of this study was to highlight the burden of breast cancer with an emphasis on AYAs in Nigeria and its implications.

**Methods:**

A retrospective review of data from cancer registries in Nigeria between 2009 and 2016 was carried out.

**Result:**

s. Among AYA females in Nigeria, breast cancer was by far the most common cancer, constituting 50% of all cancers and 51% (2798 of 5469) of all breast cancer cases. IA third (30.8%) of breast cancer cases in all centers studied were AYAs.

**Conclusion:**

The high proportion of AYA with breast cancer is an important feature suggesting that urgent actions are required to ensure early detection and improve breast cancer care among this age group.

## 1. Introduction

Cancer occurs on all body sites, in both genders and across all ages. Globally, breast cancer (BC) was the most common cancer worldwide (excluding nonmelanomatous skin cancers) in 2020 based on GLOBOCAN figures, with death rates higher in low -medium income countries (LMICs) [1]. It is a leading cause of cancer morbidity and mortality among women globally. In recent times, female breast cancer has surpassed lung cancer as the most commonly diagnosed cancer, with an estimated 2.3 million new cases annually [1].

Although it occurs in women of all races, however, a disparity exists in diagnosis, mortality, and survival [2]. For example, African American (AA) women have a 42% higher breast cancer death rate compared to White women despite recent advancements in management of the disease [3]. Among African American women, a 10-year report between 2000 and 2010 indicated that breast cancer mortality increased from 30.3% to 41.8% and that at the advanced stage, 5% of breast cancers are detected among White women compared to 8% of breast cancers among Black women (Yedjou et al., 2019). Recent findings indicate higher deaths in transitioning versus transitioned countries (15.0 vs 12.8 per 100,000) [4]. It is worrisome to note that while incidence in the African region (although rising) was comparatively lower than in other continents except Asia, its age-standardized mortality rate ranked the highest globally. Nigeria, the most populous African nation, has the highest mortality rate [5]. Globally, BC is uncommon in women less than 40 years of age, occurring only 4–6% [6]. It is the most common malignancy in this age group. However, a significant increase has been observed in recent times among premenopausal women [6]. Adolescents and young adults (AYAs) are defined by the National Cancer Institute, USA, to be age 15 to 39 years [6, 7]. Breast cancers in women less than 40 years of age are more likely to be clinicopathologically characterized by higher histological grade, higher rates of Her2/neu overexpression, lower estrogen receptor (ER) positivity, and more lymph node positivity [8].

In addition, the diagnosis at more advanced stages of the disease contributes to a less favorable prognosis compared with older women. Adolescents and young women with breast cancer have special psychosocial issues such as concerns for fertility preservation and effects on family life and career. Special attention is therefore essentially required for this group. Young women are generally treated similarly to older patients [8], but given the biologic, host, socioeconomic, and psychosocial differences, their BC care should differ.

### 1.1. Overview of Breast Cancer in Nigeria

The estimated population of Nigeria in 2020 by the World Bank is 206,139,587 million people occupying a total surface area of approximately 923,768 square kilometers [9]. Close to 31% of the population in 2012 were youths aged 15-35 years [10]. In Nigeria, high mortality from breast cancer persists owing to inadequate population awareness, poor health seeking behavior, low levels of female education, and empowerment in addition to a poor health system leading to suboptimal treatment services [11]. Although like in many Sub-Saharan Africa (SSA), the incidence of breast cancer appears to be relatively low; however, survival in BC is generally poor amidst other competing public health challenges [2]. An intriguing aspect however is that aside from Asia, Africa has the highest age-standardized mortality rate compared to other continents with higher incidence. Nigeria has been reported to have the highest breast cancer mortality rate [5]. With a prevalent rate of 69.1 per 100,000 and mortality rate of 6.23 per 100,000 based on data from Institute of Health Metrics Evaluation (IHME) (https://vizhub.healthdata.org/gbd-compare/ accessed on July 15, 2022), it has one of the worst outcome of breast cancer globally.

In addition, the stage at diagnosis is a major contributing factor to poor survival from breast cancer [12]. Stage at diagnosis is a major determinant of survival from breast cancer. Early-stage disease is associated with a better prognosis than late-stage disease. In most high-income countries (HICs), an earlier stage of diagnosis and therapeutic advances are major contributors to the sharp reductions in breast cancer mortality rates over the past two decades [13]. In a published review, the percentage of late-stage breast cancer at diagnosis in Black populations from sub-Saharan Africa in 2010 was higher than in Black and White populations in the USA 40 years earlier [12].

In Nigeria, data on the proportion of female AYAs with breast cancer is scarce. Furthermore, there has been little improvement in the outcome of breast cancer in this age group over the years. There is a need to determine the proportion of AYA with breast cancer in the country to guide the development of an agenda towards providing relevant care targeted at this population to improve survival. The objective of this study was to determine the profile of adolescents and young adults with breast cancer in Nigeria by examining current Nigerian data on breast cancer among females and to do a literature review on the peculiar problems faced by breast cancer patients in this age group which is distinct from disease in older ones (40 years and above).

## 2. Materials and Methods

**Figure 1:**
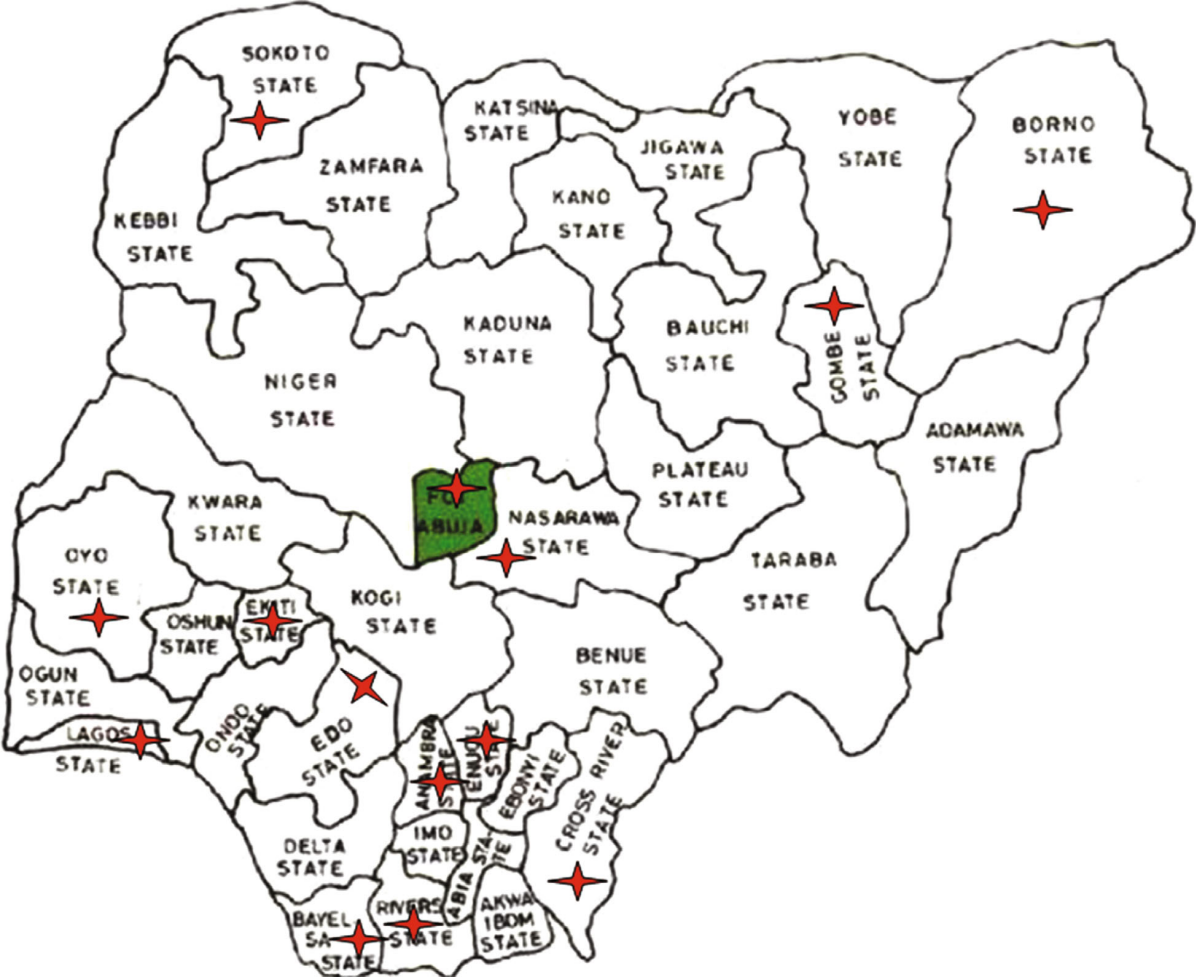
Map of Nigeria showing the location of 15 cancer registries that contributed data to this report (adapted from cancer in Nigeria 2009-2016 [14]).

We obtained cancer incidence data from the February 2021 version of the Nigerian National System of Cancer Registries data on Cancer in Nigeria [14]. This is the most comprehensive data on cancer incidence in Nigeria and was compiled using available data from dynamic population and hospital-based cancer registries in Nigeria. The publication was from the Nigerian Federal Ministry of Health. The publication contains information on cancer cases diagnosed from 2009 to 2016 based on reports from available cancer registries in Nigeria. The locations of the registries that contributed data that were used in this analysis are presented in Figure 1.

Data on breast and all cancers among AYAs were extracted.

This publication is from the reanalysis of the above data. We also reviewed data from the Ibadan Cancer Registry Report for 2009 to 2012 published in 2016 [15]. This is the oldest population-based cancer registry in Nigeria. We report here the number of new cancer cases for the most recent seven years of available data (2009–2016) with focus on AYA. Incidence rates were available for 2009-2016, and we examined overall trends and geographical distribution.

## 3. Results

There were 27 cancer registries in Nigeria in the report. Data from 15 cancer registries in Nigeria from 2009 to 2016 were analyzed. The remaining cancer registries did not have sufficient data entries on their pages. Eight out of the selected 15 registries were population-based while 7 were hospital-based. The proportion of cancer in persons aged 15-39 years in descending order is indicated in Table 1.

The total number of cancer cases recorded was 5624. Breast cancer was the most common cancer in the age group, accounting for nearly half of all cases of cancer in females, followed by cancer of the uterine cervix.

The number of breast cancer cases among all age groups in each of the cancer registries in Nigeria is presented in Table 2.

A total of 9149 cases were reported during the period. Out of these, 2798 cases were aged 15 to 39 years. The proportion of cases among this age group ranged from 23.73% to 42.47% in the various centers.

**Figure 2:**
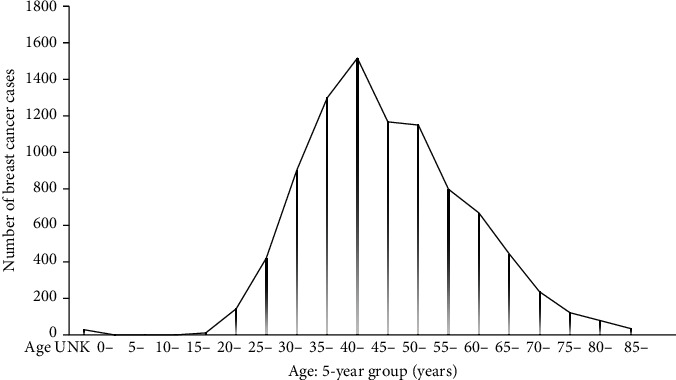
Age distribution of breast cancer in females across various age groups in 15 centers in Nigeria.

The age-specific incidence of breast cancer in females from the data is represented in Figure 2.

The age group most affected by breast cancer in Nigeria is the 40-44-year group.

Breast cancer among female adolescents and young adults (15-39 years) in 15 cancer registries in Nigeria from 2009 to 2016 is shown in Table 3.

The total number of cases reported was 2798, representing 30.58% of all breast cancer cases in the report.

The geographical spread of breast cancer between Southern and Northern Nigeria is presented in Table 4.

More cases of breast cancer were recorded in the South (7034) than in the North (2188). The number of cases among adolescents and young adults was also more in the South. However, breast cancer was the most common cancer recorded in most registries. The exception is the Federal Medical Centre, Keffi, Nasarawa State, in the North where cervical cancer was slightly higher than breast cancer (28.6%; 88 cases for cervical cancer followed by breast cancer 27.9%; 86 cases) and Sokoto State (also in the North) with cancer of the cervix (age-standardized rate, ASR = 15.3 per 100,000; 36 cases) followed by breast cancer (ASR = 12.0 per 100,000; 42 cases).

## 4. Discussion

There is a substantial proportion of young people affected by cancer even though the number is less than in older counterparts. However, the impact of cancer in AYAs is great due to the premature morbidity and death associated with such occurrences [6]. The focus of this study was to determine the magnitude of the problem of breast cancer among adolescents and young adults in Nigeria as breast cancer is the most common form of cancer in the country. The distribution of cancer in these age groups varies according to region although the incidence is not uniformly reported especially in sub-Saharan Africa. The global burden of cancer among AYAs was reported to be 1.19 million new cases with 396000 deaths in 2019. This is equivalent to ASR of 59.6 new cancer cases per 100 000 people per year. The highest ASR of 14.2 per 100,000 person-years was recorded in low- middle income countries [16]. In this report, the total number of cancer cases recorded among persons aged 15 to 39 years was 5624, with breast cancer being the most reported cases (2798), constituting about 50% of all cancer cases followed by cancer of the uterine cervix (Table 1). This is more than the figure in the USA where even though breast cancer is the most common cancer in this age group, but the proportion is about 30% of all cancers [6, 17]. This data is similar to a global report where breast cancer was reported to be the most common cancer among adolescents and young adults, followed by cancer of the uterine cervix, although with variable proportions [16].

The age-standardized incidence of breast cancer shows a steady rise from the adolescent period till it peaks at the 40–44-year age group. It starts declining but with a second lower peak at 46-50 years age group (Table 2). A study from Northern Thailand reported the peak age of breast cancer at 50-54 years [18].

Among all breast cancer patients in Nigeria during the study period, the proportion of AYAs aged between 15 and 39 years with breast cancer was 30.58% (range 24-42%) across different centers (Tables 2 and 3). This is higher than the proportion from USA in which breast cancer in this age group accounts for 5.6% of all invasive breast cancer in women [6]. In South Korea, data from the South Korea Central Cancer Registry showed that breast cancer in this age group accounts for about 9% of the total breast cancer cases in the data base [19]. An institutional report from Northern India found 33% of breast cancer among patients less than 40 years. This figure is similar to the findings from this report. In addition, the highest breast cancer incidence among all breast cancer cases was between the age group 40-44 years. Although this age group is not considered a young adult, the late presentation of the breast may suggest that the onset of the disease was in the range sequel-35-39 years.

Thus, the proportion of breast cancer among young persons in Nigeria is higher than what obtains in high income countries. It has been reported that among Blacks in the United States, the proportion of breast cancer among females aged less than 40 years is higher than among their White counterparts [20]. The high proportion of cancer in this age group in this study may be due to environmental, genetic, ethnic, and lifestyle factors which need to be further explored.

The age-specific incidence for female breast cancer in the data increased rapidly until the age 35 years group. It then continued to increase at a slightly slower rate with a peak at age 40 years group before it gradually declined. (Figure 2). This does not follow the phenomenon called Clemmensen's hook in the age-specific incidence of female breast cancer observed in developed countries apart from Japan [21]. This phenomenon describes a typical age incidence pattern of breast cancer, which has a rapid rise until age 50 years (around menopause), followed by a slower rate of increase until age 65 years before it starts to decline. The first peak at 50years is considered promoted by ovarian hormonal changes while the second peak being 65 years is considered promoted by increased adrenal hormonal activities, obesity and comorbidities such as hypertension and diabetes. [22]. The point of inflection on the curve at age 50 is described as Clemmensen's hook [23]. This pattern is attributed to the bimodal distribution with a first peak of early onset of the disease followed by a second peak with a later age of onset of the disease [24]. The phenomenon of Clemmensen's hook is interpreted to be due to the overlapping of two curves corresponding to pre- and post-menopausal tumors, respectively [25]. The pattern seen in this report demonstrates more younger patients with breast cancer among the study population. The first peak is at age 40 years – younger than among Caucasians. The pattern also reflects the higher proportion of breast cancer among younger age groups compared with their Caucasian counterparts. The absence of the second (higher) peak may be associated with low life expectancy in Nigeria [55 years in women] [26] with lower aged population who may not live long enough to develop breast cancer compared with populations of developed countries. Furthermore, the sharp rise at age 35 years and the younger age of the first peak at 40 years (a decade lower than among Caucasians) may have implications on the timing of preventive and screening activities in the Nigerian population. Screening may need to start at an earlier age than among Caucasians and other populations. To this effect, although the earliest age of commencement of mammogram screening in some guidelines is 40 years [27, 28] ultrasound (USS) based screening may need to be recommended for the general population at age 35 to spare the radiation effects of mammogram and the possible difficulties with mammogram in dense breast pattern which is preponderant in this younger age group. Breast MRI could be offered in suspicious or unsatisfactory findings while mammogram can be done as a diagnostic procedure in highly suspicious cases. In individuals with a strong family history of breast cancer, screening can be commenced earlier than age 35 years. It is worthy to note that the lack of effective screening method for breast cancer in this age group may contribute to late presentation.

Although breast cancer was the most common cancer recorded in most of the registries, however, while this was correct for all registry entries in Southern Nigeria, two centers in Northern Nigeria had cervical cancer as the most frequent cancer followed by breast cancer. This may reflect geographical variations in cancer incidence in the country. This may be as a result of differences in climate, environmental factors, culture, dietary, and other social patterns that exist between the two regions. These factors are known to have influence on the incidence of breast cancer generally [29]. The proportions of AYAs with breast cancer in the northern and southern regions were similar at 32.2% and 31.5%, respectively (Table 4). This implies that similar patterns of incidence of breast cancer are seen in the entire country. This is comparable with a report from the USA where little evidence of variation in breast cancer among the different states was reported [30].

### 4.1. Limitations of the Study

The study used existing data with possibilities of incompleteness. Some data were from hospital-based registries while some were population-based registries. The hospital-based records might not reflect the incidence in the populations represented. The number of registries in the southern parts of the country were more than in the North (10 vs 5). The data captured from the North might be incomplete. Nevertheless, this report is the most comprehensive data on the incidence of breast cancer among AYAs aged 15-39 years in Nigeria.

## 5. Conclusion

AYAs with cancer face peculiar challenges following cancer diagnosis. Breast cancer was the most common cancer diagnosed among persons aged 15 to 39 years, constituting about 50% of all cancers in that age group. Among all breast cancer patients, the proportion of those aged 15-39 years was 30.58%. There was no regional variation in breast cancer incidence among AYAs between the northern and southern parts of Nigeria. The high proportion of AYA with breast cancer is an important feature suggesting that urgent actions are required to address the specific need for improved breast cancer care among adolescents and young adults in Nigeria.

## Figures and Tables

**Table 1:** Proportion of cancer sites in females aged 15-39 years from 2009 to 2016 in descending order (*N* = 5624).

Order	Sites	Total no. of cases	Percent
1	Breast	2798	49.75%
2	Cervix uteri	412	7.33%
3	Ovary	355	6.31%
4	Other skin	152	2.70%
5	Connective and soft tissue	151	2.68%
6	Non-Hodgkin lymphoma	125	2.22%
7	Liver	118	2.10%
8	Kaposi sarcoma	98	1.74%
9	Bone	97	1.72%
10	Corpus uteri	90	1.60%
11	Uterus unspecified	92	1.64%
12	Rectum	85	1.51%
13	Colon	75	1.33%
14	Thyroid	65	1.16%
15	Nasopharynx	60	1.07%
16	Hodgkin disease	59	1.05%
17	Eye	52	0.92%
18	Kidney	43	0.76%
19	Nose, sinuses, etc.	43	0.76%
20	Myeloid leukemia	42	0.75%
21	Other female genital organs	38	0.68%
22	Vulva	37	0.66%
23	Anus	37	0.66%
24	Salivary glands	34	0.60%
25	Placenta	32	0.57%
26	Brain, nervous system	28	0.50%
27	Stomach	27	0.48%
28	Vagina	25	0.44%
29	Mouth	19	0.34%
30	Bladder	18	0.32%
31	Pancreas	18	0.32%
32	Trachea, bronchus, and lung	17	0.30%
33	Melanoma of skin	12	0.21%
34	Hypopharynx	11	0.20%
35	Tongue	10	0.18%
36	Esophagus	10	0.18%
37	Lymphoid leukemia	10	0.18%
38	Myeloproliferative disorders	10	0.18%
39	Leukemia unspecified	9	0.16%
40	Other thoracic organs	8	0.14%
41	Lip	7	0.12%
42	Pharynx unspecified	6	0.11%
43	Multiple myeloma	5	0.09%
44	Adrenal gland	4	0.07%
45	Other endocrine	4	0.07%
46	Small intestine	4	0.07%
47	Gallbladder, etc.	4	0.07%
48	Tonsil	4	0.07%
49	Larynx	2	0.04%
50	Mesothelioma	2	0.04%
51	Renal pelvis	2	0.04%
52	Other oropharynx	2	0.04%
53	Immunoproliferative diseases	1	0.02%
	Total	5624	100

**Table 2:** Breast cancer in females across all ages in 15 registries in Nigeria.

																					
	All ages	Age UNK	0-	5-	10-	15-	20-	25-	30-	35-	40-	45-	50-	55-	60-	65-	70-	75-	80-	85-	(%)
Abuja 2009-2016 (FCT) Federal Capital City)	1661	23	0	0	0	0	39	98	208	270	295	204	207	125	74	56	33	19	7	3	45.3
Benin 2014-2016 Edo State	434	0	0	0	0	0	6	15	31	51	72	61	64	51	32	29	10	2	3	7	38.3
Calabar 2009-2016 Cross River State	271	0	0	0	0	1	5	22	25	44	47	38	26	24	20	7	5	4	2	1	40.3
Ekiti 2014-2016 Ekiti State	158	0	0	0	0	0	9	14	16	19	15	22	13	14	15	6	8	0	4	3	42.5
Enugu 2012-2016 Enugu State	847	0	0	0	0	4	17	39	96	119	150	126	102	68	53	41	23	5	2	2	45.8
Gombe 2009-2016 Gombe State	340	0	0	0	0	0	3	18	36	48	47	54	42	25	25	13	13	6	7	3	33.5
Ibadan 2009-2012 Oyo State	637	5	0	0	0	0	4	24	47	83	97	85	87	59	64	42	16	14	10	0	37.7
Keffi 2009-2016 Nasarawa State	86	0	0	0	0	0	0	1	13	12	22	14	6	6	6	1	1	2	2	0	27.9
LASUTH 2009-2016 Lagos state	1499	0	0	0	0	1	20	74	128	202	253	210	176	128	138	91	35	21	15	7	53.2
LUTH 2012-2016 Lagos State	1690	0	0	0	0	4	19	46	131	211	283	265	233	163	148	97	45	30	11	4	57
Maiduguri 2016 Borno State	59	0	0	0	0	0	3	2	7	7	11	8	11∗	5	2	1	2	0	0	0	39.9
NAUTH 2009-2016 Anambra State	844	0	0	0	0	1	12	37	74	125	124	126	124	81	51	39	22	15	9	4	40.9
UDUTH 2014-2015 Sokoto State	42	0	0	0	0	0	0	2	4	7	10	2	10	2	3	0	2	0	0	0	25.9
UPTH 2009-2016 Rivers State	581	0	0	0	0	1	7	30	86	91	83	78	63	46	39	22	22	5	6	2	55.3
Yenagoa 2009-2016 Bayelsa State	73	0	0	0	0	0	0	5	7	19	17	8	5	7	2	1	2	0	0	0	43.7
Total	9149	28	0	0	0	13	144	427	909	1308	1526	1175	1158	804	672	446	239	123	78	36	

LASUTH: Lagos State University Teaching Hospital, Lagos State; LUTH: Lagos University Teaching Hospital, Lagos State; NAUTH: Nnamdi Azikiwe University Teaching Hospital, Anambra State; UPTH: University of Port-Harcourt Teaching Hospital, Rivers State; UDUTH: Usman Dan Fodio University Teaching Hospital, Sokoto State; UNK: unknown.

**Table 3:** Proportion of breast cancer cases among female AYAs (15-39 years) in 15 cancer centers in Nigeria from 2009 to 2016.

	Breast cancer cases among AYA	Total breast cancer cases	Percent
Abuja 2009-2016	615	1661	37.03
Benin 2014-2016	103	434	23.73
Calabar 2009-2016	96	271	35.42
Ekiti 2014-2016	58	158	36.71
Enugu 2012-2016	275	847	32.47
Gombe 2009-2016	104	340	30.59
Ibadan 2009-2012	158	637	24.80
Keffi 2009-2016	26	86	30.23
LASUTH 2009-2016	425	1499	28.35
LUTH 2012-2016	411	1690	24.32
Maiduguri 2016	19	59	32.20
NAUTH 2009-2016	249	844	29.50
Sokoto 2014-2015	13	42	30.95
UPTH 2009-2016	215	581	37.01
Yenagoa 2009-2016	31	73	42.47
Total	2798	9149	30.58

LASUTH: Lagos State University Teaching Hospital, Lagos State; LUTH: Lagos University Teaching Hospital, Lagos State; NAUTH: Nnamdi Azikiwe University Teaching Hospital, Anambra State; UPTH: University of Port-Harcourt Teaching Hospital, Rivers State.

**Table 4:** Comparison of breast cancer incidence among female adolescents and young adults between Northern and Southern states of Nigeria.

	Breast cancer cases among AYA	Total breast cancer cases	Percent
Northern states			
Abuja 2009-2016	615	1661	37.03
Gombe 2009-2016	104	340	30.59
Keffi 2009-2016	26	86	30.23
Maiduguri 2016	19	59	32.2
Sokoto 2014-2015	13	42	30.95
Total	777	2188	
Southern states			
Benin 2014-2016	103	434	23.73
Calabar 2009-2016	96	271	35.42
Ekiti 2014-2016	58	158	36.71
Enugu 2012-2016	275	847	32.47
Ibadan 2009-2012	158	637	24.8
LASUTH 2009-2016	425	1499	28.35
LUTH 2012-2016	411	1690	24.32
NAUTH 2009-2016	249	844	29.5
UPTH 2009-2016	215	581	37.01
Yenagoa 2009-2016	31	73	42.47
Total	2021	7034	

LASUTH: Lagos State University Teaching Hospital; LUTH: Lagos University Teaching Hospital; NAUTH: Nnamdi Azikiwe University Teaching Hospital, Anambra State; UPTH: University of Port-Harcourt Teaching Hospital.
